# A Follow-Up Study of Lung Function and Chest Computed Tomography at 6 Months after Discharge in Patients with Coronavirus Disease 2019

**DOI:** 10.1155/2021/6692409

**Published:** 2021-02-13

**Authors:** Qian Wu, Lingshan Zhong, Hongwei Li, Jing Guo, Yajie Li, Xinwei Hou, Fangfei Yang, Yi Xie, Li Li, Zhiheng Xing

**Affiliations:** ^1^Department of Respiratory Medicine, Haihe Clinical College of Tianjin Medical University, Tianjin 300350, China; ^2^Tianjin Institute of Respiratory Diseases, Tianjin 300350, China; ^3^Department of Radiology, Haihe Clinical College of Tianjin Medical University, Tianjin 300350, China; ^4^Department of Prevention, Haihe Clinical College of Tianjin Medical University, Tianjin 300350, China

## Abstract

We aimed to investigate changes in pulmonary function and computed tomography (CT) findings in patients with coronavirus disease 2019 (COVID-19) during the recovery period. COVID-19 patients underwent symptom assessment, pulmonary function tests, and high-resolution chest CT 6 months after discharge from the hospital. Of the 54 patients enrolled, 31 and 23 were in the moderate and severe group, respectively. The main symptoms 6 months after discharge were fatigue and exertional dyspnea, experienced by 24.1% and 18.5% of patients, respectively, followed by smell and taste dysfunction (9.3%) and cough (5.6%). One patient dropped out of the pulmonary function tests. Of the remaining 54 patients, 41.5% had pulmonary dysfunction. Specifically, 7.5% presented with restrictive ventilatory dysfunction (forced vital capacity <80% of the predicted value), 18.9% presented with small airway dysfunction, and 32.1% presented with pulmonary diffusion impairment (diffusing capacity for carbon monoxide <80% of the predicted value). Of the 54 patients enrolled, six patients dropped out of the chest CT tests. Eleven of the remaining 48 patients presented with abnormal lung CT findings 6 months after discharge. Patients with residual lung lesions were more common in the severe group (52.6%) than in the moderate group (3.4%); a higher proportion of patients had involvement of both lungs (42.1% vs. 3.4%) in the severe group. The residual lung lesions were mainly ground-glass opacities (20.8%) and linear opacities (14.6%). Semiquantitative visual scoring of the CT findings revealed significantly higher scores in the left, right, and both lungs in the severe group than in the moderate group. COVID-19 patients 6 months after discharge mostly presented with fatigue and exertional dyspnea, and their pulmonary dysfunction was mostly characterized by pulmonary diffusion impairment. As revealed by chest CT, the severe group had a higher prevalence of residual lesions than the moderate group, and the residual lesions mostly manifested as ground-glass opacities and linear opacities.

## 1. Introduction

Coronavirus disease 2019 (COVID-19) is an acute respiratory infectious disease caused by severe acute respiratory syndrome coronavirus 2 (SARS-CoV-2). As of 22 November, there have been over 57.8 million cases and 1.3 million deaths reported globally since the start of the pandemic [1]. Despite its current containment in China, the number of global cases is still on the rise. Existing research mostly focuses on the treatment of COVID-19 [2], while much remains unknown about its prognosis, especially the pathophysiological outcome of COVID-19-induced pulmonary fibrosis. Viral pneumonia tends to induce pulmonary interstitial changes. At discharge, many patients with COVID-19 experience varying degrees of dyspnea on exertion and present with postinflammatory pulmonary fibrosis as revealed by imaging examination, with some suffering from the usual interstitial pneumonia or nonspecific interstitial pneumonia [3]. Autopsy of patients who died of Severe Acute Respiratory Syndrome (SARS) has shown that diffuse alveolar injury is the most significant pathological feature of the lungs [4]. Moreover, pulmonary function tests in SARS patients 10 years after discharge still reveal diffuse and restrictive lung injury [5], suggesting long-lasting effects of coronavirus infection on the lungs. To date, most reports on pulmonary dysfunction and imaging abnormalities of patients with COVID-19 focus on patient status at discharge, while there are a few studies on long-term follow-up during the recovery period, with the reported postdischarge follow-up lasting only 3 months at most [6]. In the present study, pulmonary function and chest computed tomography (CT) assessments were performed on patients, 6 months after discharge, to further explore the recovery status of respiratory function in discharged patients with COVID-19. This could help to gain insights into the outcome of inflammation-induced pulmonary fibrosis.

## 2. Materials and Methods

### 2.1. Subjects

**Figure 1:**
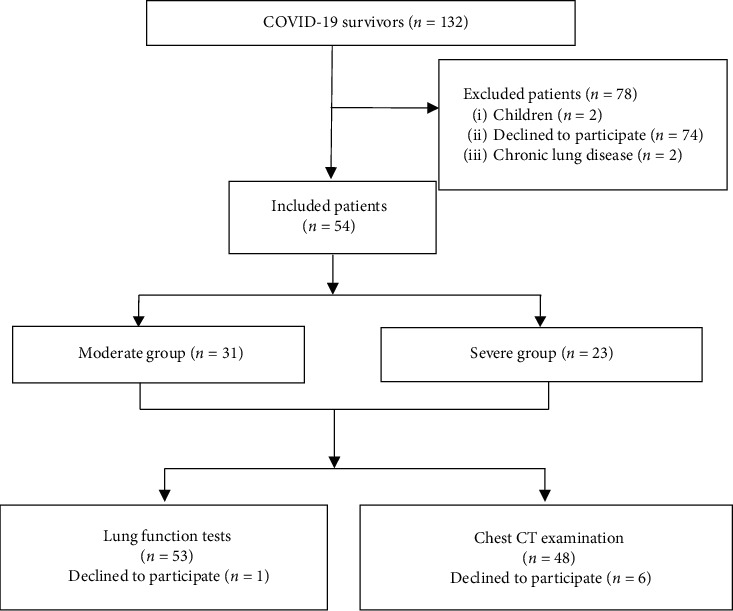
Flow chart of inclusion and exclusion criteria for patients with COVID-19 in this study. COVID-19, coronavirus disease 2019; CT, computed tomography.

A total of 137 local COVID-19 patients were admitted to Tianjin Haihe Hospital, China, between January 21, 2020, and September 1, 2020; of these, five were deceased by June 2020, leaving 132 patients for screening. After excluding two patients who were children, 74 patients who refused to undergo reexamination 6 months after discharge, and two patients with chronic lung disease (one with interstitial lung disease and the other following lung cancer resection), the present study finally enrolled 54 patients. The 54 patients consisted of 22 females (40.7%) and 32 males (59.3%), with an average age of 47 (interquartile range, 36.8–57.3) years. The subjects were divided into two groups: a moderate group and a severe group. The moderate group included patients with a fever, respiratory tract symptoms, and pneumonia based on imaging. The inclusion criteria for the severe group were as follows: (1) shortness of breath (respiratory rate, ≥33 times/min), (2) mean resting oxygen saturation ≤93%, (3) arterial partial pressure of oxygen/fractional inspired oxygen concentration ≤300 mmHg (l mmHg = 0.133 kPa), or (4) lung imaging showing that the lesion had progressed significantly (>50%) within 24–48 hours. Thirty-one (57.4%) and 23 (42.6%) patients were included in the moderate and severe groups, respectively. The inclusion criteria were patients who met the COVID-19 diagnostic criteria developed by the Ministry of Health of the People's Republic of China [7], with diagnosis confirmed at the Tianjin Center for Disease Control and Prevention using nucleic acid testing. The exclusion criteria were as follows: (1) patients whose follow-up and physical examination data were missing, (2) patients with chronic lung disease, or (3) patients with mental illness who were unable to participate. Of the 54 patients, one declined to undergo pulmonary function tests due to tracheotomy, while six refused to undergo imaging examination (Figure 1).

### 2.2. Methods

#### 2.2.1. Collection of General Information

Patient names, sex, age, symptoms, and comorbidities were collected by means of questionnaires in conjunction with the review of electronic case records.

#### 2.2.2. Chest CT Examination

Retrospective analysis of the chest CT data of 48 patients (29 and 19 in the moderate and severe group at admission, respectively) at the peak of hospitalization and 6 months after discharge was performed. Chest CT scans were performed on all patients using a Canon 64-slice helical CT scanner (Aquilion Prime 128, Canon Medical Systems, Otawara, Japan). The acquired chest CT images were interpreted by three radiologists. The frequency of CT findings was counted according to the Fleischner Society's chest CT findings classification criteria [8]. In the event of inconsistency in interpretation among the three radiologists, clinical data and examination results were discussed in a group discussion until a consensus was reached and a conclusion was made. Moreover, a semiquantitative visual scoring method [9] was used to score the CT images of each single lung lobe according to the area percentages of the lesions in the single lung lobe. A lung lobe without lesions was scored as 0, while a lung lobe with lesion area percentages of <25%, ≥25% to <50%, ≥50% to <75%, and ≥75% were scored as 1, 2, 3, and 4, respectively. The total score of the five lobe categories ranged from 0 to 20. Each CT image was reviewed and scored by three radiologists independently, and the scores were averaged to represent the final score of the CT image.

#### 2.2.3. Pulmonary Function Tests

At 6 months after discharge, pulmonary function tests were performed using Master Screen Body (Jaeger ms-pft analysis unit, Würzburg, Germany) and according to the American Thoracic Society standards [10] on the following items: forced vital capacity (FVC), forced expiratory volume in one second (FEV1), FEV1/FVC ratio, maximum expiratory flow at 50% FVC (FEF50), maximum expiratory flow at 75% FVC (FEF75), maximum midexpiratory flow (MMEF75/25), and diffusing capacity for carbon monoxide (DLCO) corrected for hemoglobin. Except for FEV1/FVC, all other pulmonary function tests were measured as a percentage of the predicted value. Measured FVC <80% of the predicted value indicated restrictive ventilatory dysfunction. When two or three of the measured FEF50, FEF75, and MMEF75/25 were <65% of the predicted values, the patients were diagnosed as having small airway dysfunction. Measured DLCO <80% of the predicted value indicated pulmonary diffusion impairment.

### 2.3. Statement of Ethical Approval

This study was approved by the Ethics Review Committee of Haihe Clinical College of Tianjin Medical University (approval no. HHYY-202001, no. 2020xkz-02, and no. 2020xkm03). All patients signed a written informed consent form.

### 2.4. Statistical Methods

Statistical analyses were performed using SPSS Statistics for Windows, version 26.0 (IBM Corp., Armonk, NY, USA). Measurement data following a normal distribution were expressed as mean ± standard deviation (*x* ± *s*), and between-group comparisons were subjected to independent sample *t*-tests. Data not following a normal distribution were expressed as median (25^th^ percentile, 75^th^ percentile). Count data were expressed as percentages (%) and compared using *χ*^2^ test. Statistical significance was defined as *p* < 0.05.

## 3. Results

### 3.1. Basic Information

Five patients were excluded from the present study due to death. These patients were all elderly, and their basic characteristics are shown in Table 1. A total of 54 patients who had recovered from COVID-19 were enrolled, including 17 (31.5%) with comorbidities, namely, 10 cases of hypertension (18.5%), seven cases of diabetes (13.0%), and three cases of coronary heart disease (5.6%). The average age of patients in the severe group was 54.4 ± 13.6 years, which was significantly higher than that of those in the moderate group (43.3 ± 15.0 years). The proportion of patients with diabetes was significantly higher in the severe group (26.1%) than in the moderate group (3.2%). The demographic characteristics and clinical symptoms of the 54 patients are summarized in Table 2.

At follow-up examination 6 months later, the main symptoms among the patients were fatigue (13/54; 24.1%), exertional dyspnea (10/54; 18.5%), and smell and taste dysfunction (5/54; 9.3%), with no significant differences between the two groups. The average age of those in the severe group (54.4 ± 13.6 years) was significantly higher than that (43.4 ± 15.0 years) of the moderate group. The main comorbidity was hypertension (10/54; 18.5%), followed by diabetes. Specifically, the prevalence of diabetes in the severe group was significantly higher than that in the moderate group (26.1% vs. 3.2%).

### 3.2. Pulmonary Function

Twenty-two patients (41.5%) presented with pulmonary dysfunction. Four patients (7.5%) presented with abnormal FVC, indicating restrictive ventilatory dysfunction. Ten patients (18.7%) presented with small airway dysfunction, and 17 (32.1%) with pulmonary diffusion impairment characterized by DLCO <80% of the predicted value. Pulmonary diffusion impairment was the most common pulmonary dysfunction in these patients. The between-group comparison of pulmonary function is presented in Table 3.

### 3.3. Pulmonary High-Resolution CT

**Figure 2:**
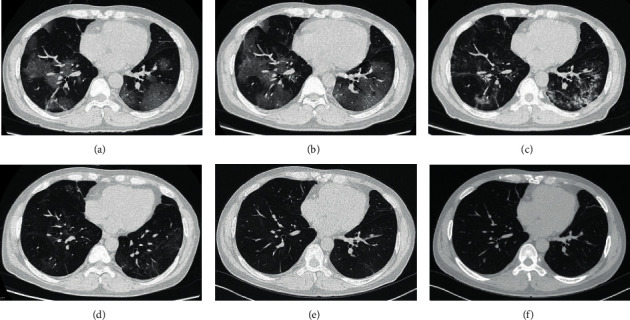
A 46-year-old male patient with severe coronavirus disease 2019. (a) On the seventh day after onset, multiple ground-glass opacities were detected in both lungs. (b) On the ninth day after onset, the distribution of ground-glass opacities in both lungs was wider than before. (c) On the 18^th^ day after onset, some lesions in both lungs became smaller and were absorbed, while the local density increased. (d) On the 34^th^ day after onset and 2 days before discharge, the lesions were significantly absorbed in both lungs. (e) One month after discharge, light ground-glass opacities and linear opacities were visible in both lungs. (f) Six months after discharge, thin linear opacities were still visible in both lungs.

Comparing the chest CT data of 48 patients with COVID-19 at the peak of hospitalization (Table 4), eleven patients (22.9%) still had abnormal CT findings 6 months after discharge (Table 5), consisting of one in the moderate group (1/29, 3.4%) and 10 in the severe group (10/19, 52.6%) (Figure 2), with a significant between-group difference. High-resolution chest CT revealed nine patients (18.8%) with the involvement of both lungs, none with the involvement of only the left lung, and two (4.2%) with the involvement of only the right lung. Specifically, four patients (8.3%) had involvement of the right upper lobe, six (12.5%) the right middle lobe, 10 (20.8%) the right lower lobe, five (10.4%) the left upper lobe, and nine (18.8%) the left lower lobe. The severe group not only had a higher proportion of patients with abnormal CT findings involving both lungs than in the moderate group, but it also had a higher proportion of patients with involvement of a given lung lobe. Ground-glass opacities (10/48, 20.8%), linear opacities (7/48, 14.6%), and pleural thickening (5/48, 10.4%) were common high-resolution CT findings in these patients, especially in the severe group. Semiquantitative visual scores of both lungs, the right lung, and the left lung were all higher in the severe group than in the moderate group (*p* < 0.05) (Table 6).

## 4. Discussion

The present results showed that patients in the severe group were older than those in the moderate group. The five deceased patients who were hospitalized in our research center were all elderly. The United States Centers for Disease Control and Prevention reported that COVID-19 death was associated with age ≥65 years [11]. The present results showed that the proportion of severe patients with diabetes was significantly higher than that of moderate patients. Carsana et al. [12] proposed that when treating severe patients, clinicians not only need to consider diabetes control but also need to balance glucose-lowering therapy with the treatment of viral infection. Taken together, the above findings suggest that it is necessary to consider the follow-up and control of diabetes in the postdischarge management of patients with COVID-19.

This study showed that fatigue and exertional dyspnea were the most common symptoms in COVID-19 patients 6 months after discharge. Pulmonary function tests revealed that pulmonary diffusion impairment was the most common pulmonary dysfunction. An autopsy study of lung tissues from 38 deceased COVID-19 patients showed that the pathological changes in the patients' lungs were characterized by diffuse alveolar injury, hyaline membrane formation, interstitial edema, and type 2 alveolar epithelial cell hyperplasia [13]. Persistent lung injury and pulmonary dysfunction in patients with COVID-19 are suggestive of such pathological changes. Similar pathological changes have also been observed in survivors of other viral pneumonias. A follow-up study of 383 SARS patients reported that 27.3% had pulmonary diffusion impairment at discharge. Specifically, 40 patients who had pulmonary diffusion impairment were subjected to one-year follow-up, and 20 patients still had pulmonary dysfunction one year after discharge [14]. As suggested above, it is necessary to conduct pulmonary function tests, especially pulmonary diffusion tests, in patients 6 months after discharge.

Six months after discharge, the proportion of patients with residual lung lesions was significantly higher in the severe group than in the moderate group, indicating that the lesions were absorbed more rapidly in the moderate group than in the severe group during the recovery period. The severe group had a higher proportion of patients with involvement of both lungs than the moderate group 6 months after discharge. Furthermore, the severe group had a higher proportion of patients with involvement of a given lung lobe—the right upper lobe, the right middle lobe, the right lower lobe, the left upper lobe, or the left lower lobe. Xie et al. [13] analyzed the chest CT findings of 99 patients with COVID-19 and observed that 25% of the patients presented with unilateral lung lesions and 75% with bilateral lung lesions, consistent with the present findings. The present CT findings of residual lesions in the two groups of patients were mainly ground-glass opacities, followed by linear opacities. Ground-glass opacities and linear opacities may be attributed to lesions that remained in the interstitial tissue, such as in the alveolar wall, due to the absorption of intra-alveolar exudate. The follow-up CT findings were subjected to semiquantitative visual scoring based on the proportion of patchy ground-glass opacities and lung consolidations. The results showed that the scores of the left lung, the right lung, and both lungs were all significantly higher in the severe group than in the moderate group, suggesting that the severe group had higher area proportions of ground-glass opacities and lung consolidations in the lung lobes than those in the moderate group, and, thus, would require a longer recovery time.

There is currently no literature on the 6-month follow-up of patients with COVID-19. The present study evaluated the clinical, pulmonary function, and imaging characteristics of patients with COVID-19 during rehabilitation. However, this study has several limitations. First, the number of patients enrolled in the study was small. The number of local COVID-19 patients in Tianjin, China, was relatively small and most patients had fully recovered during the short-term follow-up and refused to participate in further follow-up. Second, of the 54 patients enrolled, one was unable to undergo pulmonary function tests due to tracheotomy and six refused chest CT examination, resulting in missing data. Third, COVID-19-induced lung injury should be subjected to long-term observation. However, the patients enrolled in the present study only came for follow-up at 6 months after discharge. The follow-up should continue over a longer period of time.

## 5. Conclusions

The pulmonary dysfunction due to SARS-CoV-2 infection improved over time, but affected patients did not fully recover 6 months after discharge. After 6 months of recovery, abnormal lung CT findings and pulmonary dysfunction were still observed in some COVID-19 patients and, especially, imaging abnormalities were observed more frequently in severe patients. These observations suggest that patients with COVID-19 may suffer from persistent lung injury after viral infection and, thus, should be subjected to long-term follow-up.

## Figures and Tables

**Table 1:** Basic information and symptoms of the deceased patients.

Characteristic		Severe (*n* = 5)
Sex, *n* (%)	Male	2 (40)
	Female	3 (60)

Age (years)		66.4 ± 4.23

Comorbidity, *n* (%)	Hypertension	3 (60)
	Type 2 diabetes	3 (60)
	Coronary heart disease	4 (80)

The number of days from onset to death	—	32 ± 38.4

**Table 2:** Basic information and symptoms of the 54 patients.

Characteristic		Moderate (*n* = 31)	Severe (*n* = 23)
Sex, *n* (%)	Male	18 (58.1)	14 (60.9)
	Female	13 (41.9)	9 (39.1)

Age (years)		43.4 ± 15.0	54.4 ± 13.6

Comorbidity, *n* (%)	Hypertension	5 (16.1)	5 (21.7)
	Type 2 diabetes	1 (3.2)	6 (26.1)
	Coronary heart disease	0 (0.0)	4 (17.4)

Symptom, *n* (%)	Exertional dyspnea	4 (12.9)	6 (26.1)
	Fatigue	6 (19.4)	7 (30.4)
	Cough	2 (6.5)	1 (4.3)
	Sore throat	3 (9.7)	0 (0.0)
	Smell and taste dysfunction	1 (3.2)	4 (17.4)
	Nausea	1 (3.2)	2 (8.7)
	Loss of appetite	0 (0.0)	4 (17.4)
	Abdominal pain and diarrhea	1 (3.2)	2 (8.7)

**Table 3:** Comparison of pulmonary function between moderate and severe patient groups.

	Moderate (*n* = 31)	Severe (*n* = 22)	*t*	*p*
TLC (%)	97.8 ± 11.8	99.5 ± 11.7	−0.525	0.602
FVC (%)	97.8 ± 11.8	99.5 ± 11.7	−0.525	0.602
FEV1 (%)	96.6 ± 13.5	98.5 ± 11.5	−0.547	0.587
FEV1/VCMAX%	102.6 ± 6.7	103.2 ± 4.1	−0.435	0.665
FEF50 (%)	89.1 ± 22.9	90.2 ± 19.2	−1.77	0.86
FEF75 (%)	70.9 ± 29.8	73.9 ± 21.3	−0.405	0.687
MMEF (%)	82.1 ± 23.5	83.7 ± 17.0	−0.255	0.800
DLCO-SB (%)	83.7 ± 11.2	85.1 ± 12.1	−0.425	0.673

DLCO-SB, diffusing capacity for carbon monoxide corrected for hemoglobin; FEF50, maximum expiratory flow at 50% FVC; FEF75, maximum expiratory flow at 75% FVC; FEV1, forced expiratory volume in one second; FVC, forced vital capacity; MMEF, maximum midexpiratory flow; TLC, total lung capacity; VCMAX%, maximum vital capacity percentage.

**Table 4:** Comparison of chest CT between moderate and severe patients at the peak of hospitalization.

	Moderate (*n* = 29)	Severe (*n* = 19)	*χ* ^2^	*p*
Abnormal CT findings of both lungs	24	17	3.601	0.165
Abnormal CT findings of the right lung only	4	2		
Abnormal CT findings of the left lung only	1	0		

Affected lung lobes				
The right upper lobe	18	19	7.326	**0.007**
The right middle lobe	8	16	14.722	**<0.001**
The right lower lobe	23	17	0.279	0.598
The left upper lobe	18	17	4.365	**0.037**
The left lower lobe	26	15	0.372	0.542

Ground-glass opacities	27	19	—	0.512
Consolidation opacities	21	14	0.009	0.923
Linear opacities	10	15	9.094	**0.003**
Reticular opacities	18	17	4.365	**0.037**
Traction bronchiectasis	15	12	0.610	0.435
Pleural thickening	8	8	1.089	0.297

CT, computed tomography.

**Table 5:** Comparison of chest CT between moderate and severe patients 6 months after discharge.

	Moderate (*n* = 29)	Severe (*n* = 19)	*χ* ^2^	*p*
Abnormal CT findings	1	10	13.058	**<0.001**
Abnormal CT findings of both lungs	1	8	8.866	**0.003**
Abnormal CT findings of the right lung only	0	2	—	0.152
Abnormal CT findings of the left lung only	0	0	—	—

Affected lung lobes				
The right upper lobe	0	4	4.189	**0.041**
The right middle lobe	0	6	7.778	**0.005**
The right lower lobe	1	9	10.895	**0.001**
The left upper lobe	0	5	5.932	**0.015**
The left lower lobe	1	8	8.866	**0.003**

Ground-glass opacities	1	9	10.895	**0.001**
Consolidation opacities	0	0	—	—
Linear opacities	0	7	9.726	**0.002**
Reticular opacities	0	3	2.561	0.110
Traction bronchiectasis	0	1	—	0.396
Pleural thickening	0	5	5.932	**0.015**

CT, computed tomography.

**Table 6:** Semiquantitative visual score of lung lesions in moderate and severe patients.

Group	Score of the right lung lesions	Score of the left lung lesions	Overall score of both lung lesions
Moderate (*n* = 29)	0 (0, 0)	0 (0, 0)	0 (0, 0)
Severe (*n* = 19)	1 (0, 2)	0 (0, 2)	1 (0, 3)
*Z*	−3.959	−3.377	−3.938
*p*	**<0.001**	**0.001**	**<0.001**

## Data Availability

The data used to support the findings of this study are available from the corresponding author upon request.
